# Dentistry in State Institutions

**Published:** 1899-11

**Authors:** 


### DENTISTRY IN STATE INSTITUTIONS.

Upon another page Dr. Catching, of Atlanta, Ga., announces the appointment of a dental surgeon to the Georgia Insane Asylum. While it is believed that this is the first official appointment to a State institution of this kind, it is by no means the first recognition of the value of dentistry in other institutions. It is presumed that in all the large and many of the smaller charitable homes there are to be found regularly appointed dentists. It is so in Philadelphia in the House of Refuge, Girard College, Deaf and Dumb, Institution of the Blind, etc.

While this is true, and a striking evidence of the appreciation of dental surgery, and a marked advance over former years, there has been a singular want of appreciation by the legislative body of the State of the need of the inmates of insane asylums and prisons. No appointments of dentists have been regularly made to these institutions. There have been for brief periods dentists called in to the State Insane Asylum, but it has been through the intelligent appreciation of the superintendent.

If the State authorities failed to provide medical attendance for the inmates under their care, they would justly be regarded as negligent, and would be held accountable to an indignant public sentiment; but here are large bodies of people left entirely without aid, and every dentist can appreciate what that means in suffering and deterioration of health.

The writer has had some experience in insane institutions, and of the embarrassments of those in charge when unable to relieve their patients. That these would welcome the appointment of a skilled dentist needs no assertion here. With no means at hand to relieve suffering, resort must be had to the forceps in extreme cases, but there is much that the forceps will not relieve and where it should never be used, intelligent treatment being substituted.

The time has come for dentists to assert themselves and demand recognition for their own specialty, not only in the army and navy, but everywhere in all State institutions, and the several State societies should begin work at once with the legislators of the States, and demand that this be done. Georgia has taken the initiative; now let other States follow this excellent example, and have dentists appointed to all institutions under the control of the government. Good policy and humanity demand this, and if the facts be carefully laid before our legislators, it is believed that the great value of these appointments would be speedily recognized. It only needs a concerted and vigorous effort to accomplish it.

